# Linking Microbial Functional Gene Abundance and *Daqu* Extracellular Enzyme Activity: Implications for Carbon Metabolism during Fermentation

**DOI:** 10.3390/foods11223623

**Published:** 2022-11-13

**Authors:** Yu-Ting Zhang, Yu-Ke Deng, Yong-Fang Zou, Bao-Lin Han, Ji-Zhou Pu, Jia-Quan Rao, Dan Huang, Hui-Bo Luo

**Affiliations:** 1 College of Bioengineering, Sichuan University of Science and Engineering, No. 180, Xueyuan Street, Huixing Road, Zigong 643000, China; 2 Sichuan Tuopai Shede Liquor Co., Ltd., Suining 629209, China; 3 Brewing Biotechnology and Application Key Laboratory of Sichuan Province, Yibin 644000, China

**Keywords:** *Daqu*, extracellular enzymes, carbohydrates, metagenomics

## Abstract

*Daqu* is the starter of *Baijiu*, it provides the microbes and enzymes necessary for fermentation. Studies have already established carbohydrate metabolism as the primary functional module in *Daqu* fermentation. The present study investigated the changes in microbial functions and the relationship between carbohydrate metabolism-related functional genes and extracellular enzyme activity during the *Daqu* fermentation. Amplicon sequencing identified 38 bacterial and 10 fungal phyla in *Daqu* samples, while shotgun metagenomic sequencing classified and annotated 40.66% of the individual features, of which 40.48% were prokaryotes. KEGG annotation showed that the pathways related to metabolites were less in the early fermentation stage, but higher in the middle and late stages. The functional genes related to pyruvate metabolism, glyoxylate and dicarboxylate metabolism, and propanoate metabolism were relatively high in the early and late stages of fermentation, while that for start and cross metabolism was relatively low. The study also found that amino sugar and nucleoside sugar metabolism were dominant in the middle stage of fermentation. Finally, the correlation network analysis showed that amylase activity positively correlated with many carbon metabolism-related pathways, while liquefaction activity negatively correlated with these pathways. In conclusion, the present study provides a theoretical basis for improving and stabilizing the quality of *Daqu.*

## 1. Introduction

*NongXiang Baijiu* is a Chinese distilled spirit produced through traditional solid-state fermentation. It is one of the most consumed alcoholic beverages in the world, with a consumption of more than 10 billion liters per year and a history of over hundreds of years. Unlike other famous spirits, such as whisky, vodka, and brandy, *NongXiang Baijiu* uses a unique starter called *Daqu*, made primarily from wheat and peas through a unique fermentation process. Under open conditions, microorganisms from *Daqu* raw materials and the production environment grow and metabolize, and the temperature and humidity of fermentation are controlled by opening or closing doors and windows, as well as uncovering or covering straw. These microorganisms provide enzymes for degrading the macromolecular substances in the raw materials, flavor substances, and their precursors. Thus, *Daqu* is a crucial factor that determines *Baijiu*’s quality.

Liquefaction, saccharification, and protease activity are important indicators of *Daqu* quality. Liquefaction and saccharification are characterized by the degradation of starch and the production of reducing sugars, respectively. They reflect the ability of *Daqu* to hydrolyze macromolecular carbohydrates, such as starch, cellulose, and lignin, in raw materials during *Baijiu* production. Meanwhile, the protease activity reflects the ability of *Daqu* to degrade protein into amino acids. Several studies have reported that the plant compound content (phenols and flavonoids) and biological activity significantly improve during the fermentation of *Daqu* [1,2]. These changes occur due to various microbial hydrolases, such as β-glucosidase, α-amylase, cellulase, and protease, which effectively act on the cell wall components [3]. In plants, most phytochemicals are bound to cell wall components, such as cellulose, hemicellulose, and lignin [4], through the ether, ester, or glycosidic bonds, and exist in insoluble forms in grains, which are difficult to extract. During fermentation, microorganisms (especially fungi) secrete enzymes to hydrolyze these ether, ester, or glycosidic bonds that effectively decompose the cell wall structure and release the insoluble form of the compounds [3], improving the bioactivity of *Daqu* products. At the same time, numerous monosaccharides also provide a material basis for the growth of brewing microorganisms and the synthesis of ethanol. Thus, the *Daqu* production enzyme system accumulated by microorganisms during *Daqu* production is closely related to the formation of *Baijiu* flavor substances and ethanol production, which are affected by microbial diversity and environmental factors [5].

In the past decade, the microbial communities and hydrolase activity of different types of *Daqu*, such as *Jiang*, *Nong*, and *QingXiang*, have been characterized using traditional and molecular methods. Moreover, during the production of *NongXiang Daqu*, environmental parameters directly influence the microbial community structure of *Daqu* and indirectly affect the production of enzymes by affecting microbial activity [6]. Currently, sequencing ribosomal amplicons are used to elucidate the microbial community composition of *Daqu* [7]. However, the amplified microbial community map does not provide genomic or functional details of the microbiome [8]. The research emphasis on *Daqu* microorganisms should also include the metabolic functions of specific microbial communities. Understanding the macrogenome of the microbiota is necessary to classify and obtain a given community’s genomic and functional details. Meanwhile, studies have proven that carbohydrate metabolism is the most critical functional module in *Daqu* fermentation [9]. Almost all flavor compounds in *Daqu*, such as alcohols, esters, phenols, and acids, are derived from carbohydrate metabolism during fermentation [10,11], which is closely related to the growth and material exchange of microorganisms in the fermentation environment. Therefore, a comprehensive analysis of the functional genes related to carbohydrate metabolism is necessary to understand the microbial succession and activities during *Daqu* fermentation.

Furthermore, researchers have found that the differences in microbial communities among different types of *Daqu* are related to environmental conditions [7]. For example, water and acid stress lead to the transformation of microbial structure in the thermophilic stage [12]. Specifically, the temperature during *Daqu* production is an important factor affecting microbial community composition. Studies have also shown that the activity of enzymes is closely related to environmental factors, which influence the metabolic activities of microorganisms and affect the accumulation of enzymes [13]. Therefore, we believe that there could be a neglected important part in the changes of environmental factors, enzyme activity, and microbial community; that is, the abundance of functional genes. Moreover, the association between extracellular enzyme activity and functional gene abundance [14] may determine the formation of complex metabolic compounds in *Daqu* fermentation. The genome and function of the *Daqu* microbial community have not been fully explored.

Therefore, in this study we investigated the relationship between microbial functional genes and extracellular enzyme activity during *Daqu* fermentation using shotgun metagenomic sequencing, as well as physical and chemical index analysis. The research focused on determining extracellular enzyme activity, the mechanisms affecting the functional gene abundance, and the corresponding functional microbial groups. The study’s findings will help to understand the mechanisms controlling the composition and function of microbial communities, and provide a theoretical basis for high-quality and stable *Baijiu* production.

## 2. Materials and Methods

### 2.1. Sample Collection

We collected *Daqu* samples from a *NongXiang Baijiu* enterprise in Suining, Sichuan, China (30°10′–31°10′ N, 105°03′–105°59′ E). The samples were collected from the same position of the fermentation room at 0, 3, 6, 16, 20, and 29 days of *Daqu* fermentation, and labeled as D0, D3, D6, D16, D20, and D29, respectively. These samples were ground separately and stored at 4 °C for physicochemical and enzyme analyses and at −80 °C for DNA extraction.

### 2.2. Extracellular Enzyme Properties of Daqu

The saccharification and liquefaction activities were measured following previously reported methods [14]. The saccharification activity was calculated as the milligrams of soluble starch converted into glucose by one gram of *Daqu* in one hour at 35 °C and a pH of 4.6. The liquefaction activity was calculated as the gram of liquefied starch obtained from one gram of *Daqu* in one hour at 35 °C and a pH of 4.6.

### 2.3. DNA Extraction, Amplicon Sequencing, and Shotgun Metagenomic Sequencing

Total DNA was extracted from the *Daqu* samples following the cetyltrimethylammonium bromide (CTAB) extraction method [15], with slight modifications; three DNA samples were extracted from each *Daqu* sample. The concentration of the extracted DNA was detected with a NanoDrop2000 ultramicro spectrophotometer, and the purity and integrity were analyzed by 1% agarose gel electrophoresis (AGE). The high-quality DNA samples were stored at −80 °C until further use.

For the amplicon library preparation, the amplification of 16S and ITS2 DNA fragments was performed using the common amplified primers and methods of prokaryotic 16S rDNA V3-V4 region (338F and 806R) and fungal ITS region (ITS1F and 2043R). The purified amplicons were pooled at equimolar concentration, and analyzed by paired-end sequencing on an Illumina NovaSeq PE250 platform (Illumina, San Diego, CA, USA) according to the standard protocols described by Majorbio Bio-Pharm Technology Co., Ltd. (Shanghai, China).

For metagenomic library preparation, the DNA was sonicated to obtain 350 bp fragments. These DNA fragments were end-repaired, 3′-adenylated, and amplified using Illumina (Illumina, San Diego, CA, USA) sequencing adapter-specific primers. After quality control, the DNA libraries were quantified and normalized, and 150 and 250 bp paired-end reads were generated on the Illumina HiSeq4000 and MiSeq (Illumina, San Diego, CA, USA) platforms according to the manufacturer’s instructions with slight modifications. More than 10 Gb of clean data and 30,000 clean reads were generated for each metagenomic and amplicon sample. The amplicon sequence variants (ASV) with single base accuracy were obtained by sequence correction using the DADA2 algorithm. The raw reads were deposited into the NCBI database (Accession Number: PRJNA892894).

### 2.4. Amplicon Data Processing

The raw reads were demultiplexed, quality-filtered using fastp (v0.20.0) (https://github.com/OpenGene/fastp, accessed on 2 June 2022) [16], and merged using FLASH (v1.2.7) (https://ccb.jhu.edu/software/FLASH/index.shtml, accessed on 4 June 2022) [17]. Sequences were then clustered into operational taxonomic units (OTUs) at a 97% similarity level [8,18] using UPARSE version 7.1 (http://www.drive5.com/uparse, accessed on 6 June 2022) [8]. Meanwhile, the chimeric sequences were identified and removed. The taxonomy of each representative OTU sequence was analyzed using RDP Classifier (v2.2) [19] against a 16S rRNA database (Silva v138) and an ITS rRNA database (unite 8.0) using a confidence threshold of 0.7.

### 2.5. Metagenomic Data Processing

The raw reads from the metagenome sequencing were used to generate clean reads by removing adaptor sequences and low-quality reads (reads with N bases, a default length of 40 bp, and a default quality threshold ≤38) using Readfq (v8, https://github.com/cjfields/readfq v8, https://github.com/cjfields/readfq, accessed on 18 June 2022). The high-quality reads were then assembled to contigs using MEGAHIT [20] with default parameters (–min-count 2 -k-min 27 –k-max 87 –k-step 10), using Bowtie2 software (v2.2.4) (https://bowtie-bio.sourceforge.net, accessed on 22 June 2022). The clean data of every sample were compared with their scaftigs to obtain the unused paired-end reads. The parameters [21,22] used for statistical analysis were as follows: –end-to-end, -sensitive, -I 200, -X 400, and a filter fragment shorter than 500 bp in all of the scaftigs.

Then, the unigenes were compared to the functional databases, including the KEGG (v2018-01-01, http://www.kegg.jp/kegg, accessed on 26 June 2022) the eggNOG (v4.5, http://eggnogdb.embl.de/#/app/home, accessed on 30 June 2022), and the CAZy (v201801, http://www.cazy.org/, accessed on 5 July 2022), using DIAMOND software (v0.9.9.110, https:/github.com/bbuchfink/diamond/, accessed on 10 July 2022) with the following parameters: blastp, -e le-5. The best BLAST hits were selected for subsequent analysis.

### 2.6. Statistical Analysis

Furthermore, to compare the richness and diversity of microbials in *Daqu* samples, alpha diversity indexes, such as Chao1, Shannon, and Simpson indexes, were calculated using Qiime [23]. The β diversity was analyzed using principal coordinate analysis (PCA) based on R studio (v3.2.5)( https://www.r-project.org/, accessed on 5 July 2022). Heatmaps of gene abundance and pathway changes were plotted in R with the pheatmap package. Finally, the correlation network of pathways and enzymes was constructed using Cytoscape (v3.7.0) (https://cytoscape.org/, accessed on 27 July 2022).

## 3. Results

### 3.1. Composition of the Microbial Community in Fermented Daqu

We performed both amplicon (16S rDNA and ITS2) and deep shotgun metagenomic sequencing using the *Daqu* samples. Initially, we obtained 307,726 valid 16S rRNA sequences and 386,276 valid ITS sequences. We obtained 64,379 effective fungal sequences and 51,287 bacterial sequences from the six samples. The total and clean reads obtained for each sample are shown in S1 File. Moreover, our DNA extraction protocol yielded high molecular weight DNA suitable for shotgun metagenomic sequencing. The method generated 134,566.83 Mbp shotgun metagenomic sequences and an average of 12,233.35 Mbp paired-end reads (150 bp) from all samples. After quality control, the total amount of data and the average amount of data were 133,928.27 Mbp and 12,175.30 Mbp, respectively, and with an effective data quality of 99.53%. The average length of scaffolds was 2013.13 bp, the maximum length was 771,669 bp, the N50 was 10,490.83 bp, and the N90 was 696.83 bp. Meanwhile, the average length of scaftigs was 1933 bp, the N50 was 7325 bp, and the N90 was 834 bp. The total length of ORFs (Open Reading Frames) was 271.92 Mbp, the average length of ORF was 586.53 bp, and the GC content was 53.59%, among which 2,65,147 were complete genes, accounting for 57.19% of the total number of non-redundant genes (unigenes).

**Figure 1:**
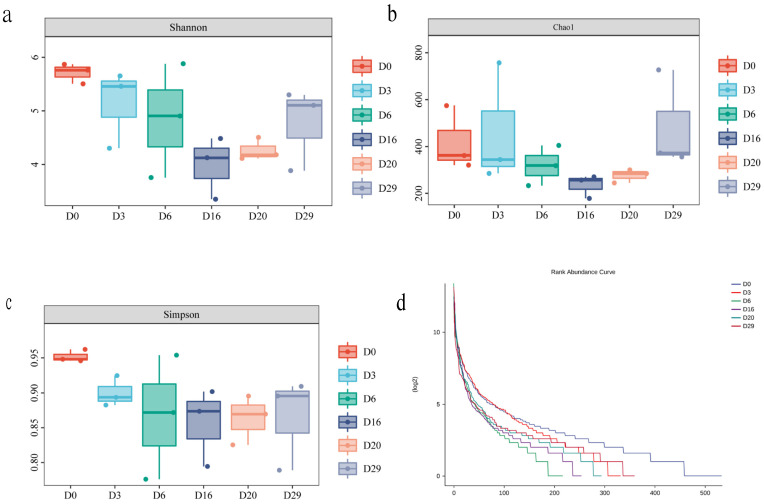
Changes of alpha diversity index of microbial community during *Daqu* fermentation. Shannon index (**a**), Chao1 index (**b**), Simpson index (**c**), Rank Abundance Curve (**d**).

Then, we evaluated the taxonomic characteristics and relative abundance of the microbial communities at the genus and species levels during the different stages of fermentation (D0, D3, D6, D16, D20, and D29) using the shotgun metagenomic sequencing data (Figure 1). At all stages of *Daqu* fermentation, viruses and archaea accounted for less than 0.4% and 0.2% of the specified readings. On D0, D3, D6, D16, D20, and D29 of fermentation, bacteria accounted for 96%, 95%, 75%, 92%, 36%, and 82% of the reads and fungi accounted for 0.6%, 4%, 25%, 8%, 64%, and 18% of the reads. Thus, the relative abundance of bacteria was higher than that of fungi; however, the abundance of fungi, especially some thermophilic ones, rapidly increased during the fermentation’s middle and late stages.

**Figure 2:**
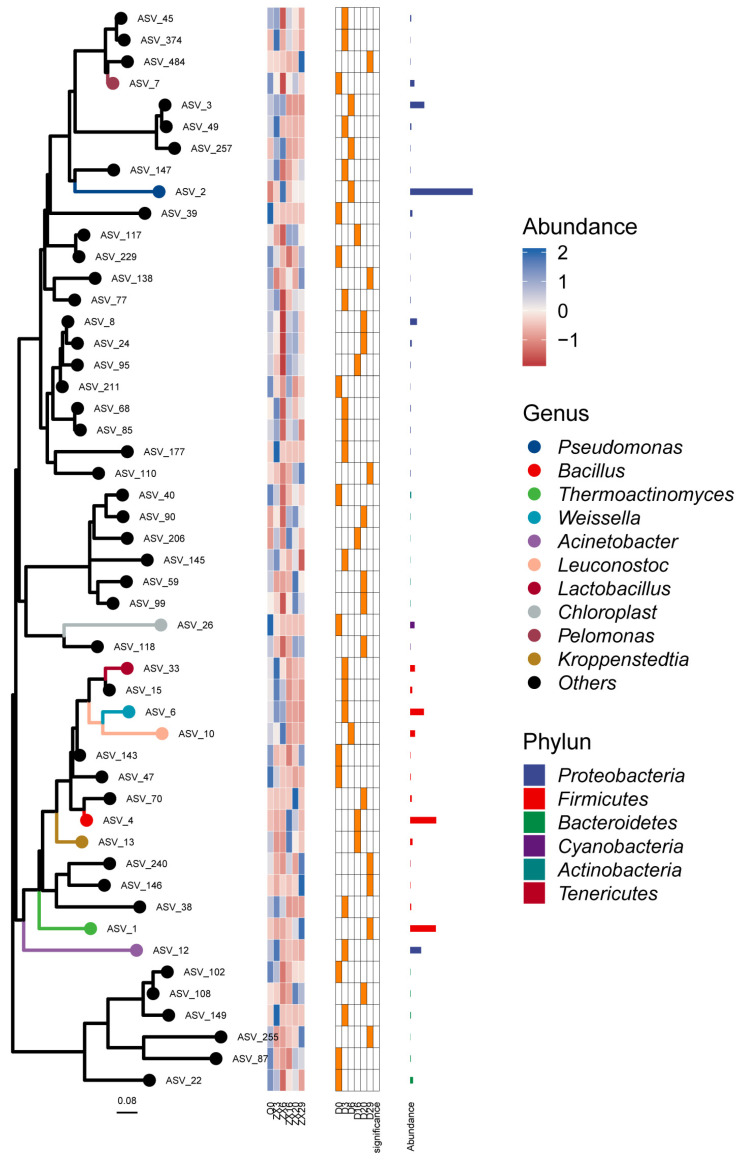
Taxonomic phylogenetic tree and species abundance heatmap of *Daqu* microorganisms (on the left is the phylogenetic tree constructed based on ASV, and in the middle is the abundance and significance of species at ASV level) (*p* < 0.05).

**Figure 3:**
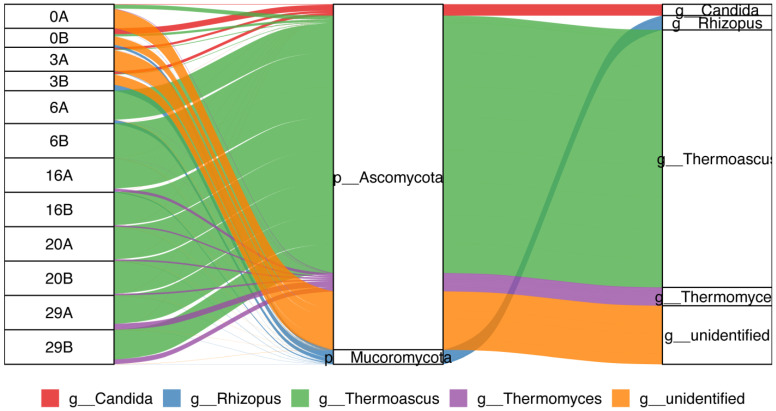
Sankey network diagram of fungal community composition in *Daqu* (based on amplicon sequencing).

Further analysis of the species composition of ASV sequences amplified by 16S rRNA of each sample showed that *Proteobacteria*, *Firmicutes*, *Bacteroidetes*, *Cyanobacteria*, and *Actinobacteria* were the dominant bacterial phyla, and *Pseudomonas*, *Bacillus*, *Thermoactinomyces*, *Weissella*, *Acinetobacter*¸ *Leuconostoc*, and *Lactobacillus* were the dominant bacterial genera. Then, according to the analysis results, the classification evolutionary tree, species abundance heat map (showing changes in the abundance of dominant species), and difference heat map (showing the group with the highest abundance of top species, and testing the significance of the difference between this group and the remaining samples. Using Wilcoxon rank sum test, take the *p*-value < 0.05 corrected by FDR as significant) (Figure 2). Meanwhile, we found no significant difference in the alpha diversity indexes of fungal species based on the ITS data of *Daqu* at different fermentation stages. *Ascomycota*, *Mucoromycota*, *Basidiomycota*, *Mortierellomycota*, and *Zoopagomycota* were the main fungal phyla; *Thermoascus*, *Rhizopus*, *Thermomyces*, *Pichia*, *Alternaria*, and *Aspergillus* were the main fungal genera. *Thermoascus* was the most predominant fungal genus, with an average relative abundance of more than 85% in the middle and late stages of fermentation (Figure 3).

**Figure 4:**
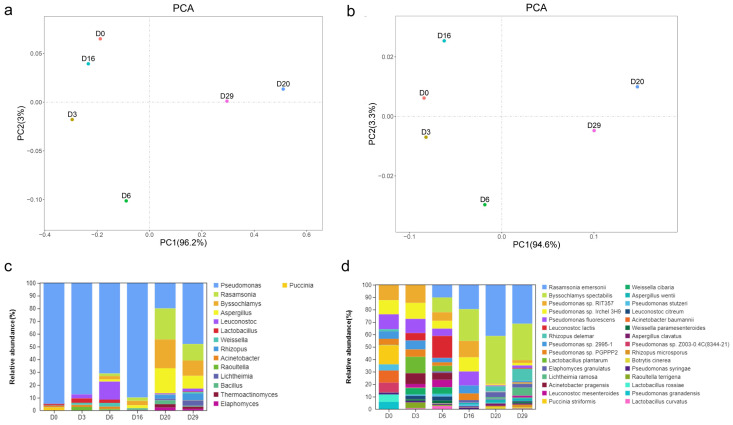
Species distribution and composition during *Daqu* fermentation stages. PCA analysis at genus level (**a**), species level (**b**), different color dots represent different samples, species composition at genus level (**c**), species level (**d**).

We then combined the amplicon sequencing and shotgun sequencing results and conducted PCA on the relative abundance of species in each sample. The results showed that, based on Bray-curtis, the two axes of genus level accounted for 96.2% and 3%, and the two axes of species level accounted for 94.6% and 3.3%. The samples of different stages were obviously separated, indicating significant differences in microbial composition across the stages of *Daqu* fermentation (Figure 4). Interestingly, at the genus level, the separation between samples D0 and D16 was small, indicating that the composition of microbial communities in these two *Daqu* samples were only slightly different (Figure 4a), and at the species level, the composition of microbial communities of different *Daqu* samples were considerably dissimilar (Figure 4b).

### 3.2. Functional Characteristics of Daqu Microorganisms at Different Fermentation Stages

**Figure 5:**
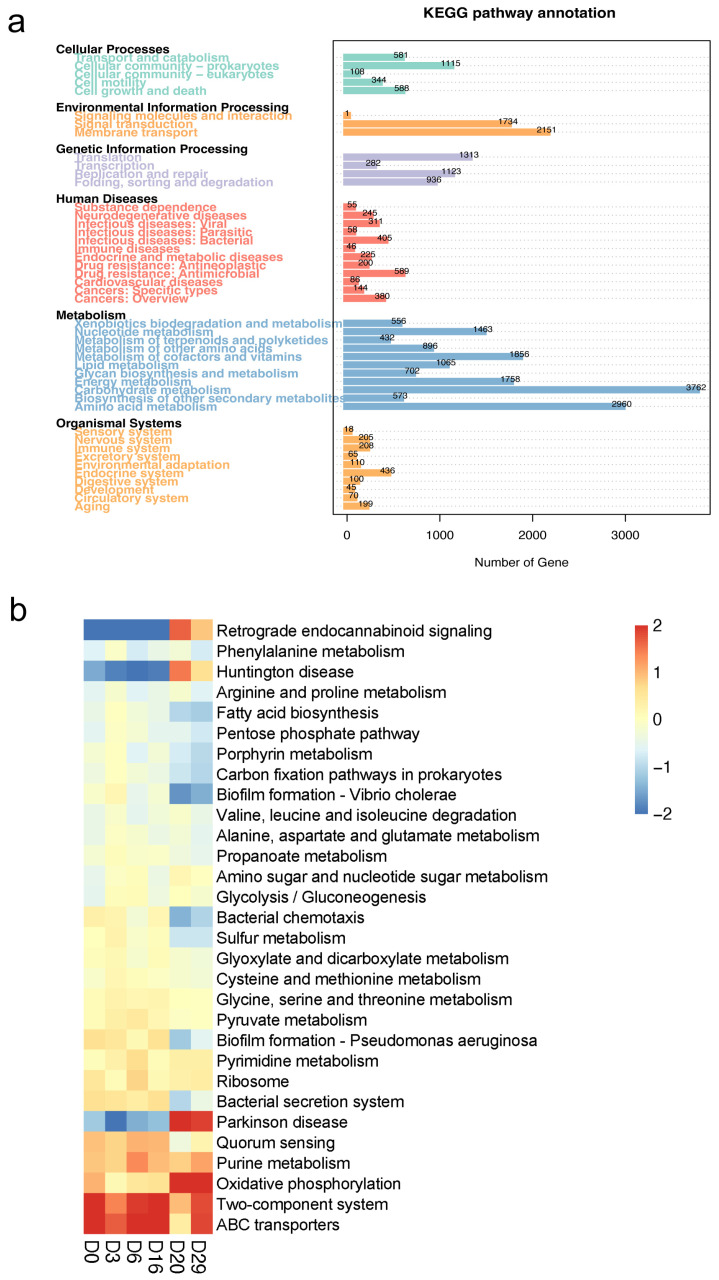
Functional changes of microbial community during *Daqu* fermentation process. Secondary pathway annotation of functional genes, the black font represents the primary metabolic pathway, and the colored fonts represent the secondary metabolic pathway. The length of the histogram indicates the abundance level of functional genes (**a**), heatmap of abundance of major metabolic pathways (*p* < 0.05) (**b**). (Base on KEGG database).

We then annotated the microorganisms based on shotgun sequencing results and the number of unigenes in the KEGG database to identify the function of *Daqu* microorganisms. Firstly, we obtained statistics on the secondary channels with non-zero abundance of unigenes and drew a chart (Figure 5a). The results showed that the types of channels annotated with human diseases and metabolism were the most abundant, and there were at least three secondary channels annotated by environmental information processing. Meanwhile, more unigenes were annotated in the two secondary channels of carbohydrate and amino acid metabolism, with 3762 unigenes in the carbohydrate metabolism category. Then, we annotated the three-level pathways to understand the specific functions of the *Daqu* microorganisms. We then determined the KO (KEGG Orthology) of the top 30 relative abundances and drew a heatmap (Figure 5b). The results showed that the abundance of ABC transporters related to cell growth was higher in the early stage of *Daqu* fermentation. However, with the progress in fermentation, oxidative phosphorylation, pyrimidine metabolism, Glycolysis/gluconeogenesis, and material metabolism-related pathways gradually become dominant. Their abundances were low in the early stages of *Daqu* fermentation, and generally became enriched in the middle and late stages, which reflects that cell growth is superior in the early stage of fermentation, and microorganisms begin frequent metabolic activities after having a certain material basis in the late stage, producing a lot of metabolites. Finally, we paid close attention to carbohydrate metabolism enriched with the largest number of unigenes (Figure 5a). The results showed that the number of functional genes of pyruvate metabolism, glyoxylate and dicarboxylate metabolism, and propanoate metabolism were relatively abundant in the early and late stages of fermentation, while the number of functional genes of start and cross-metabolism were scarce. Meanwhile, functional genes for amino sugar and nucleoside sugar metabolism were relatively more prevalent in the middle stage of fermentation.

**Figure 6:**
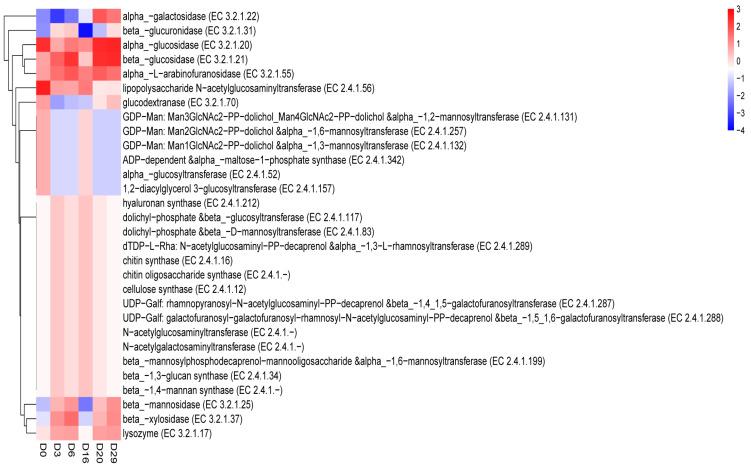
Cluster heatmap of functional abundance during *Daqu* fermentation (base on CAZy database) (*p* < 0.05).

Then, we used the metagenomic data in the carbohydrate-active enzyme database (CAZymes, http://www.cazy.org/, accessed on 7 June 2022), which has resources regarding enzymes that synthesize or decompose complex carbohydrates and glycoconjugates. It is based on the similarity of amino acid sequences in protein domains, and carbohydrate active enzymes are classified into different protein families. The database provides the classification and relevant information of enzymes associated with carbon synthesis, metabolism, and transport. It divides the main enzymes into six modules: glycoside hydrolases (GHS), glycoside transferases (GTS), polysaccharide lyases (PLS), carbohydrate esterases (CES), carbohydrate-binding modules (CBMs), and enzymes with auxiliary activities (AAS). We first annotated *Daqu* samples to level 1. In each sample, the module with the highest abundance was GH, and that with the lowest abundance was PL. Meanwhile, the abundance of various modules in unfermented samples was less than that in the fermented samples, which gradually increased with fermentation time. The heatmap shows that we clustered the enzymes with abundance from the top 30. The results indicated that alpha-glucosidase (EC 3.2.1.20) and beta-glucosidase (EC 3.2.1.21) had the highest abundance, ADP-dependent and alpha-maltose-1-phosphate synthase (EC 2.4.1.342) alpha-glucosyltransferase (EC 2.4.1.52) had the lowest abundance. Meanwhile, galactosidase (EC 3.2.1.22) was the enzyme with the lowest abundance in unfermented *Daqu* samples; this enzyme’s abundance gradually increased with fermentation (Figure 6). However, beta-glucuronidase (EC 3.2.1.31) experienced a noticeable fluctuation in fermentation progress, and gradually increased from 0-6 days. The abundance of beta-glucuronidase rapidly decreased from 6 to 16 days, attained the lowest on the 16th day, and then increased gradually from 16 to 29 days. The abundance of the functional genes related to carbon metabolism directly affects the production of metabolites, which is important for *Daqu* fermentation quality. The abundance of functional genes may be related to extracellular enzyme activity.

### 3.3. Changes in Enzyme Activity

**Figure 7:**
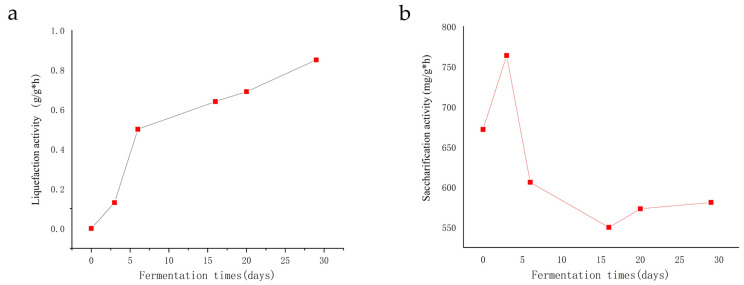
The evolution of saccharification activity (**a**), liquefaction activity (**b**), during *Daqu* fermentation.

The primary function of *Daqu* is to provide the hydrolases to degrade the macromolecular substances, such as starch and protein, in raw materials into small molecules for microbial utilization and then, finally, brewing. Therefore, liquefaction and saccharification activities are important quality indexes of *Daqu*. Liquefaction activity mainly reflects α-amylase (EC.3.2.1.1) activity, which hydrolyzes α-1,4-glycosidic bonds into short-chain dextrins, oligosaccharides, and a small amount of maltose and glucose. We found that the liquefaction activity gradually increased with the progress of fermentation, and the rise in activity was more rapid in the first six days (0–0.5 g/g·h). After the sixth day, it gradually increased until the end of fermentation (0.85 g/g·h) (Figure 7a). Meanwhile, saccharification activity reflected the ability of *Daqu* to degrade starch, cellulose, and lignin in raw materials into glucose. However, the saccharification activity rapidly increased three days before fermentation (up to 764 g/g·h), then gradually decreased, and finally reached 581 g/g·h (Figure 7b)

### 3.4. Correlation between Extracellular Enzyme Activity and Functional Genes

**Figure 8:**
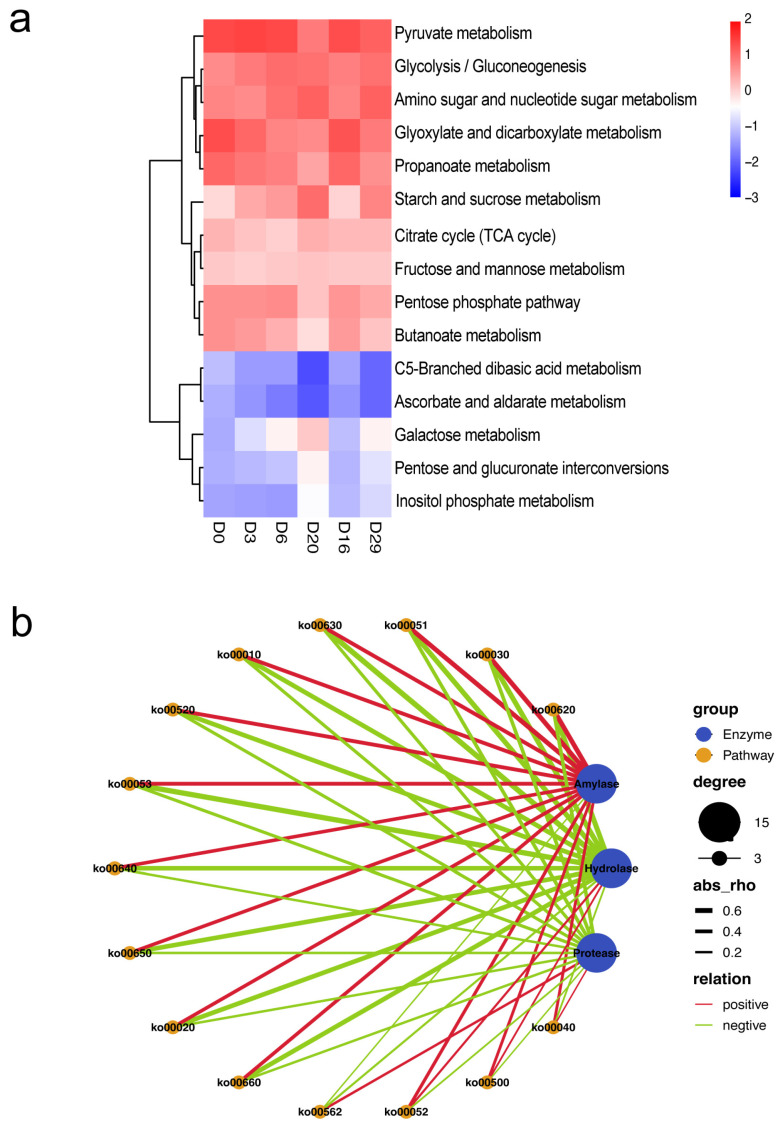
Correlation between functional genes and extracellular enzyme activity. The heatmap of functional gene abundance (*p* < 0.05) (**a**), the co-occurrence network of functional genes and carbohydrate metabolism pathways (the orange nodes represent functional genes, the blue nodes represent carbohydrate metabolism pathways, and the thickness of the lines represents the correlation strength) (*p* < 0.05) (ko00620: Pyruvate metabolism; ko00630: Glyoxylate and dicarboxylate metabolism; ko00030: Pentose phosphate pathway; ko00010: Glycolysis/Gluconeogenesis; ko00052: Galactose metabolism; ko00051: Fructose and mannose metabolism; ko00640: Propanoate metabolism; ko00520: Amino sugar and nucleotide sugar metabolism; ko00650: Butanoate metabolism; ko00660: C5-Branched dibasic acid metabolism; ko00020: Citrate cycle (TCA cycle); ko00562: Inositol phosphate metabolism; ko00052: Galactose metabolism; ko00500: Starch and sucrose metabolism; ko00040: Pentose and glucuronate interconversions) (**b**).

We finally analyzed the correlation between carbohydrate metabolism-related functional genes annotated based on the KEGG database and the extracellular enzyme activities. The results showed a positive correlation between glycoside hydrolase and the pathways related to carbohydrate metabolism during *Daqu* fermentation (Figure 8). Glycoside hydrolase hydrolyzes the α-1,4 glucoside bonds of starch from the non-reducing end to produce glucose. It does not provide raw materials for subsequent alcohol fermentation, consistent with previous studies. Meanwhile, amylase activity showed a significant positive correlation with the three pathways of ko00620 (Pyruvate metabolism), ko00030 (Pentose phosphate pathway), and ko00051 (Fructose and mannose metabolism). It showed a positive correlation with the abundance of ko00562 (Inositol phosphate metabolism) functional genes. Interestingly, glycoside hydrolase showed a significant negative correlation with these three pathways. In addition, protease negatively correlated with other pathways related to carbohydrate metabolism. Finally, the correlation network diagram (Figure 8) showed that the amylase activity positively correlated with almost all pathways related to carbon metabolism, except ko00562, but hydrolase and protease activities demonstrated an opposite association. Hydrolase and protease activities were negatively associated with the functional genes related to carbohydrate metabolism, except ko00562, ko00040 (Pentose and glucuronate interconversions), ko00052 (Galactose metabolism), and ko00500 (Starch and sucrose metabolism). In particular, a significant positive correlation was detected between hydrolase activity and ko00052 abundance.

## 4. Discussion

*Daqu* is an indispensable fermentation starter for Chinese *NongXiang Baijiu*. It provides abundant microorganisms and enzymes for fermentation during liquor production. These components play a vital role in *Daqu* quality, and *Baijiu* yield and quality [24]. In this study, we classified the potential functionals of microbial communities at three fermentation stages of *Daqu*, and determined the characteristics of *Daqu* functional genes and enzyme activities in this environment. Previous studies on *Daqu* microbial community have relied on traditional culture techniques, such as morphological and biochemical tests, which provided limited insights into microbial diversity, because 99% of microorganisms cannot be cultured [25]. Currently, studies are mainly based on amplicon sequencing without such restrictions. Moreover, due to the reduced cost of metagenomic sequencing, large-scale studies have been carried out on the microbiota in human intestines, oceans, and soils. Therefore, this study used amplicon and shotgun metagenomic sequencing to identify and characterize the *Daqu* microorganisms.

We first sequenced the bacterial 16S rRNA gene’s V3-V4 region and the fungal ITS2 region of *Daqu* samples by amplicon sequencing, and identified 7686 and 929 ASVs of bacteria and fungi, respectively. Further analysis showed that the species diversity and richness were the highest on the sixth day of *Daqu* fermentation, and the lowest for the unfermented *Daqu*. Meanwhile, the species diversity of fungi changed little during the fermentation process, and the unfermented *Daqu* had slightly lower diversity than the fermented samples at various stages. A total of 109 bacteria ASVs and 42 fungi ASVs were common during the three stages of fermentation. In addition, significant differences were detected in the species diversity of bacterial and fungal ASVs among the different time points (*p* < 0.004 and *p* < 0.009 in bacteria and fungi; Kruskal Wallis). These observations indicated the significant influence of the fermentation stage on the microbial species diversity and composition of *Daqu* samples, consistent with previous reports [26]. Amplicon sequencing identified 38 bacterial phyla and found *Proteobacteria*, *Firmicutes*, *Bacteroidetes*, *Cyanobacteria*, *Actinobacteria*, and *Tenerictes* to be the top six bacterial phyla. Previous reports also indicated *Proteobacteria*, *Firmicutes*, and *Actinobacteria* as the dominant bacteria during *Daqu* fermentation [27]. Meanwhile, *Pseudomonas*, *Bacillus*, *Thermoactinomyces*, *Weissella*, and *Acinetobacter* were the top five bacterial genera, consistent with previous reports [14]. However, at the early stage of *Daqu* fermentation, the relative abundance of *Bacillus* and *Weissella* were not among the top ten, and their abundance gradually increased with fermentation. This observation indicated conditions conducive to their growth in *Daqu* fermentation; this advantage is far greater than the impact of niche competition on them. Additionally, amplicon sequencing identified ten fungal phyla, including *Ascomycota* and *Mucoromycota*, accounting for 95.6% of the total abundance. Similar to bacteria, different varieties showed differences in the abundance of fungi at the genus level, indicating the impact of the fungal community also on the fermentation of *Daqu*. The top five fungal genera were *Thermoascus*, *Rhizopus*, *Thermomyces*, *Pichia*, and *Alternaria*, consistent with previous studies [28].

Community composition analysis based on amplicons is a classical method used for microbiome analysis. The PCR macrogenome sequence generated by the shotgun method without PCR amplification can also be used to determine the identity and relative abundance of microorganisms; however, this method does not detect ribosomal gene amplification. Due to primer deviations, it has been successfully used to interrogate different microbiomes. Therefore, this study used macrogenomic sequencing with amplicon sequencing to identify the environmental microorganisms involved in *Daqu* fermentation. Approximately 40.66% of the single features were classified and annotated, of which 40.48% were prokaryotes (bacteria and archaea; 99.55% of the total annotated single features). *Peseudomonas* was the bacterium with the highest relative abundance and was dominant at all fermentation stages. Studies have shown that *Pseudomonas* use glucose and lactose as carbon sources [29] and produce 2-keto-D-gluconic acid (2KGA) after complete glucose consumption; 2KGA is reused as an alternative carbon source to support cell growth. Thus, high carbon availability explains its dominant position in the fermentation environment. Meanwhile, *Lactobacillus* and *Leuconostoc* changed the most and demonstrated an increase first, and then a decrease. *Leuconostoc* was expressed in *Lactobacillus* in the middle stage of *Daqu* fermentation, along with high levels of genes related to pyruvate metabolism [30]. Lactic acid bacteria mainly include the facultative anaerobic microorganisms of cheese or wine, and they anaerobically grow at low pH and adapt to significant temperature changes (10–45 °C). The present study found that the genes encoding four key enzymes involved in iso-lactic acid fermentation and three key enzymes involved in iso-lactic acid fermentation were expressed. In addition, the expression of the genes encoding the four key enzymes in iso-lactic acid fermentation was much higher than that of the three key enzymes in iso-lactic acid fermentation. This phenomenon indicates that most lactic acid bacteria produce lactic acid, ethanol, and acetic acid. Studies have shown that *Lactobacillus acidophilus*, *Lactobacillus delbrueckii*, *Lactobacillus spirulina*, and *Lactobacillus salivarius* are homofermentative lactic acid bacteria [31]. In addition to these, most acid bacteria are facultative or forced heterofermentative lactic acid bacteria [32]. The lactic acid bacteria have developed various survival systems to prevent cell damage under acidic environments during fermentation [33]. In addition, different heterofermentative lactic acid bacteria showed rapid growth and effective carbohydrate metabolism. Meanwhile, among fungi, *Aspergillus* was the most abundant genus during the entire fermentation process. *Aspergillus* is a functional strain of saccharification because it produces glucoamylase and amylase, which degrade starch into fermentable sugar. Most importantly, these major active microorganisms synthesize flavor compounds that contribute to the sensory characteristics of *Baijiu*. Our research found that the fungal composition in *Daqu* at different fermentation time points was similar, indicating that the specific phenotype is the result of gene-driven selection. The change in the microbial community structure of *Daqu* also suggests that the production of *Daqu* is a natural selection process, and beneficial microorganisms get selected from complex environmental microbes for *Baijiu* production.

This study mainly revealed the potential functions of microorganisms in the *Daqu* fermentation environment, as well as the correlation between extracellular enzyme activity and functional gene abundance. Using metagenomic sequencing, we annotated the possible roles of microorganisms in the *Daqu* environment based on the KEGG database. We found that the abundance of metabolism-related functional genes was high, especially those related to carbon metabolism and amino acid metabolism because *Daqu* fermentation is spontaneous in an open environment. The complex flavor substances in *Daqu* basically come from the spontaneous metabolic activities of microorganisms [34]. We also found an enrichment of genes associated with the bacterial secretory system, bacterial chemotaxis, flagella, assembly, nutrient transporters, antibiotic resistance, and antibiotic synthesis in the regional environment, indicating the coevolution of microorganisms.

Furthermore, we obtained the changes in the abundance of functional genes encoding enzymes related to carbohydrate metabolism based on the CAZy database (Figure 7). The abundance of α-gucosidase (EC 3.2.1.20) and β-glucosidase (EC 3.2.1.21) gradually increased with fermentation time, consistent with the previous observations on these enzymes, which effectively act on cell wall components [35]. Meanwhile, α-galactosidase (EC 3.2.1.22) and β-glucuronidase (EC 3.2.1.31) decreased during fermentation. Galactosidase is the most promising glycosidase with hydrolysis and transglycosylation activities. In normal hydrolysis reactions, β-galactosidase hydrolyzes lactose and releases galactose and glucose. Due to this characteristic, β-galactosidase has been used to degrade lactose and improve fermented dairy products’ digestibility, sweetness, and flavor. In addition to the hydrolytic activity, β-galactosidase also catalyzes the transfer of sugar residues from a glycosyl donor substrate to the receptor to form new glycosidic bonds. More importantly, β-galactosidases have a wide range of substrates, enabling them to synthesize many oligosaccharides or glycosides other than gos [5]. As galactose is an important part of the glycan chain of sugar conjugates, it is involved in many biological events, such as cell recognition, communication, and microbial growth, and is closely related to the succession of the microbial community in the *Daqu* fermentation environment.

In the traditional *Daqu* making process, *Daqu* goes through a period of slight temperature rise, a period of stable high temperature, and finally, a period of cooling. Previous studies have shown that the period with the highest biomass and the most active microbial metabolic activity of *Daqu* is the first six days of fermentation (heating period), and this metabolic activity gradually weakens during the stable high-temperature period. The short-term temperature rise during the initial stage causes various extracellular enzymes in *Daqu* to slowly enter the optimal temperature, which significantly catalyzes the metabolic reactions and improves the microbial functional genes abundance [4]. Meanwhile, the continuous rise in temperature and maintenance for a long time inhibits the catalysis of enzymes and the inhibition of functional gene abundance in the regional environment, decreasing the biomass. Previous studies have found that changes in environmental factors affect the growth of microorganisms and then carbohydrate metabolism [36]. However, we believe that changes in environmental factors first affect the growth of microorganisms, and the abundance of functional genes during these changes affects the activities of various enzymes, which drive the metabolism of carbohydrates. Moore et al. (2021) provided novel insights into carbon dynamics in the fermentation environment based on the link between gene abundance and carbon-degrading enzyme activity [37]. Similarly, Guo et al. (2020) considered that the abundance of microbial functional genes for hydrolytic and oxidative carbon-degrading enzymes significantly improved the microbial processes [38]. These two examples illustrate that we can better deal with the vast microbial metagenome database by considering the microecological functional traits of *Daqu*.

Future studies should aim to elucidate a dynamic carbon model of the *Daqu* environment using the microbial genomics data. Although this study provides a comprehensive classification and partial functional analysis of microbiota in *Daqu*, research is still at an early stage. The metagenomic sequence obtained in the current study provides insights into the potential functions of the *Daqu* environment. Evaluating the interaction between microorganisms in the *Daqu* environment will help understand the expression of these characteristics, such as metatranscriptome, metaproteome, and metametametabolome, and study the culturable members of the *Daqu* fermentation environment. Moreover, a detailed understanding of the *Daqu* microbial community will help use these microorganisms to improve *Daqu* fermentation quality and productivity. Thus, the present study provides a theoretical foundation for producing high-quality *Baijiu* and promoting *Baijiu* industrial modernization.

## Data Availability

The data presented in this study are available within the article.
